# Radiographic legal age estimation based on third molar development using Machine-learning algorithms

**DOI:** 10.1371/journal.pone.0354704

**Published:** 2026-07-30

**Authors:** Noura Alsufyani, Sawsan Alowa, Hend Alrasheed, Sakher Alqahtani

**Affiliations:** 1 Department of Oral Medicine and Diagnostic Sciences, College of Dentistry, King Saud University, Riyadh, Saudi Arabia; 2 Riyadh, Saudi Arabia; 3 Department of Information Technology, College of Computer and Information Sciences, King Saud University, Riyadh, Saudi Arabia; 4 Department of Pediatric Dentistry and Orthodontics, College of Dentistry, King Saud University, Riyadh, Saudi Arabia; University of Puthisastra, CAMBODIA

## Abstract

**Background:**

Age assessment plays an important role in forensic sciences to aid in criminal or civil matters. Third molars continue developing during the legal age of adulthood. Dental age estimation based on radiographic examination is an operator-dependent procedure. The aim was to test the performance of supervised machine learning (SML) to classify individuals according to the threshold of 18 years using the third molar index (I3m).

**Methods:**

Panoramic radiographs (n = 597) of 52% males and 48% females between 13 and 26 years were used. Three Convolutional Neural Networks were compared to segment the third molar. Several machine learning algorithms were tested with a 10-fold cross-validation approach to identify the best age classification algorithm. The SML age estimation model was compared to the expert.

**Results:**

Attention U-Net achieved the highest segmentation scores, and the k-nearest neighbors (KNN) showed the best scores of classification algorithms. The SML (KNN) scored sensitivity = 77.77%, specificity = 96%, and AUC = 0.87 in males. In females, sensitivity = 77.4%, specificity = 86.7%, and AUC = 0.82. The expert scores were: sensitivity = 70.4%, specificity = 100%, AUC = 0.85 for male, and for female: sensitivity = 54.8%, specificity = 93.3%, AUC = 0.74.

**Conclusion:**

SML classified males and females below and above the legal age with high accuracy compared to manual methods. The presented work suggests that AI, using pixel-level analysis, can detect subtle cues beyond human perception. This signals a possible departure from traditional age estimation indices.

## Introduction

Chronological age is one of the key variables to be considered in anthropological and forensic studies, as well as legal procedures and migration control, where birth dates cannot be verified. Estimation methods may depend on the assessment of bone or teeth maturation. No single method outperforms others, as it depends on numerous factors. Age estimation from dental radiographs is based on the evaluation of one or more of the following: bone development of the jaws, teeth maturation, degree and eruption of crown, resorption of primary teeth, degree of open apices in teeth, volume of pulp chamber and canals, or the development and topography of the third molar.

Panoramic radiographs offer a convenient and relatively non-invasive radiographic imaging modality for estimating age. The entire dentition, maxilla, and mandible are collectively captured in a single image. There are multiple methods of age estimation in children and young adult populations using panoramic radiography; these can be atlas-based or metric. Of these, Cameriere et al [1] developed a method using the third molar maturity index (I3m) to assess the age among Caucasians. The lower left mandibular third molar (LL3rdM) was assessed in a Saudi population using (I3m) to determine if a person is younger or older than 18 years old. The cut-off value of I3m < 0.08 was useful method for estimating adulthood in both genders in several populations including the Saudi population [2,3]. Age estimation at the cut-off of 18 years serves great purposes to avoid violations of the minor’s legal rights.

Recent systematic reviews revealed that automatic approaches based on deep learning techniques in age estimation are improving in performance and time, showing a more balanced behavior, with low tendency to over- or under-estimation [4,5].

Recent studies focused on estimating age in children and adolescent populations using different methods or in different ethnicities, however few articles compared machine learning models and manual models in binary age estimation (adult vs minor at 18 years cut-off) [6].

This study aimed to utilize supervised machine learning (SML) for lower left third molar detection and extraction from panoramic dental radiographs and compare SML with human (manual) binary age estimation based on the I3m in a Saudi population.

## Materials and methods

### Data preparation and manual age estimation

A convenient sample of 600 archived panoramic dental radiographs was obtained from the Dental University Hospital, King Saud University, Riyadh, Saudi Arabia. Ethics Approval was obtained from the Institutional Review Board (IRB), Health Sciences Colleges Research on Human Subjects at King Saud University, application ID E-21–6344. Consent forms were waived, and the study was conducted in accordance with the Declaration of Helsinki. Inclusion criteria included an age range of 13–26 years, male and female, Saudi, and the presence of the left mandibular third molar. Exclusion criteria include patient motion artifact, suboptimal contrast or density, surgery, or pathology associated with the third molar. Panoramic radiographs were exported in dicom format.

The PI (expert oral and maxillofacial radiologist) applied the inclusion/exclusion criteria and performed the I3m measurements on the left third molars (two width measurements of the apices and one height measurement) using distance tools in ImageJ (Image processing and analysis in Java) [7]. To test intra- and inter-examiner reliability, the I3m measurements were completed for 30 panoramic radiographs (not included in the main study) two weeks apart, with one of the co-investigators (expert forensic dentist).

### Supervised machine learning: Molar detection and age estimation

Three convolutional neural network architectures: U-Net [8], Attention U-Net [9], and UNet++ [10] were were compiled with an Adam optimizer to minimize the binary cross-entropy loss function.

**Fig 1:**
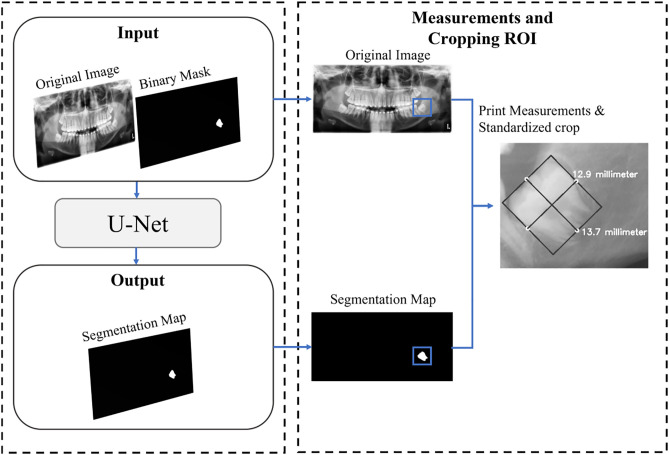
Third Molar segmentation, extraction, and measurements pipeline.

All training was set to run for 50 epochs while the early stopping technique was utilized to stop the training process when the training loss stopped improving with a batch size of 10. All radiographic images in the dataset had 2943x1435 resolution and were resized to 256x512 to reduce computational cost. Then a single binary mask was manually created using Adobe Photoshop CS5 for each image in the dataset. In each mask, the pixel value of the lower left third molar is assigned a specific value (white), whereas all other pixels are given different color (black). Third, one by one, the pre-processed images and their corresponding masks were fed to the three CNN models (Fig 1). The image dataset was randomly partitioned into 80% training set and 20% testing set to measure the performance of the trained models. A 0.5 threshold was used for convenience to avoid changing the third molar shape and size and was based on testing multiple threshold values and their overall effect. A function is created to print the measurements on the detected third molar and performed the following steps: finding the contour of the third molar, drawing the contour of the third molar, finding all contour coordinates, identifying box points around the object, drawing the corner dots of the box, calculate and draw midpoints from (right, left, up, and bottom), connect all points with lines, calculate the distance from midpoints (up, bottom) and (right, left), and the last step print the total length in millimeters on the axis.

The machine learning classification algorithms and the steps for building the age classifier:

Step 1: Data pre-processing

The independent variables were gender, apex1, apex2, height, and index. While the dependent variable is the age group (0: adult, 1: minor). Apex1, apex2, and the index have a right-skewed distribution where most of the data fall to the right while the height has a normal distribution. Apex1 is the only feature that has −1 values where some of the cases are for undeveloped third molars. Undeveloped third molars have only one apex, so the value of the apex1 was filled with −1 to distinguish these cases.

Step 2: Data splitting

Data splitting is required, so the trained model needs to be tested on new or unseen data. Therefore, the dataset was split into training and testing 80:20 respectively.

Step 3: Feature Scaling

There are different techniques of feature scaling, including normalization and standardization. Normalization bounds the values between two numbers for example [0,1] or [−1, 1]. Whereas standardization transforms the data into a variance of 1 and a mean of 0. In algorithms such as KNN, SVM, and NN, which calculate the distance between the data, if the data is not scaled, then features with the higher value will be dominant when calculating the distance; thus, all features should be scaled to weight equally. Both normalization and standardization were tested since some data features have skewed distribution while the others have a normal distribution. The result showed that both scaling methods have the same effect on the classifiers. Standardization was applied as a scaling method.

Step 4: Building Classification algorithms

The models were fine-tuned to enable achieving the best performance. One of the popular approaches to finding the best hyperparameters is grid search cross-validation. Grid search cross-validation searches for the best set of hyperparameter combinations from a grid containing hyperparameters values [11]. In addition to grid search cross-validation, the Elbow method loops over a range of k values and shows the changes in error rate and accuracy over different values of k. The optimal value of k is the value with the minimum error rate and the highest accuracy.

The last step after training the model is to predict on new data by calling the prediction function where the input is the total height, gender, apex1, apex2. The index is not required to be provided as input; it is automatically calculated. The index is the result of adding the two apices and divided by height. The result is a classification of an individual, whether minor or adult.

**Fig 2:**
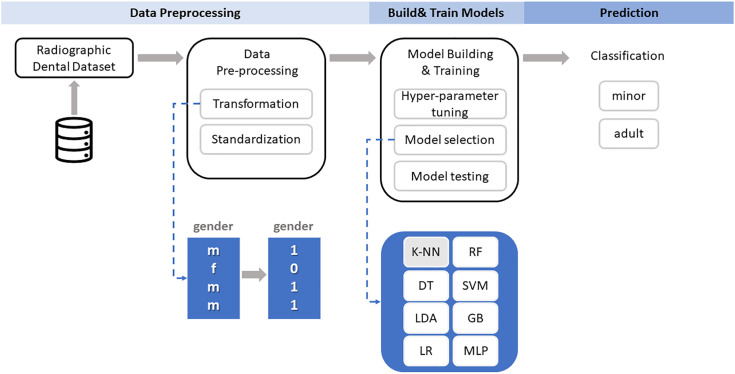
Age estimation model workflow.

The ground truth is the chronological age which is determined by subtracting the radiographic date from the individual’s birth date. The performance of the selected models was evaluated with a set of performance measures (accuracy, specificity, precision, Sensitivity, and f-measure) in addition to the confusion matrix and receiver operating characteristics (ROC). A 10-fold cross-validation is also applied to evaluate the performance of the models. Fig 2 illustrates the workflow.

K-NN was selected as a base classifier in addition to testing multiple algorithms, including RF, DT, SVM, LDA, GB, LOGR, and MLP. Chronological age (i.e., the ground truth) was encoded as adult if ≥18 years and minor if < 18. Independent variables were gender, apex1, apex2, height, and index. The dependent variable was age classification as minor vs adult based on I3M (< 0.08).

## Results

Three images were excluded from the dataset due to motion artifact and suboptimal resolution. All images have a similar field of view and resolution. There were 207 samples of minors and 390 samples of adults. The gender distribution was nearly even (52% male and 48% female).

The intra-examiner agreement for tooth measurement (I3M) was excellent (0.96 [CI 0.92–0.98]) as well as the inter-examiner agreement (0.95 [CI 0.89–0.98]).

Due to the complexity and variation of root apex anatomy, it was challenging for the model to measure root width at the developing apex. Use of landmarks was attempted, but was futile due to the large variation in tooth orientation and stage of development. As such, we proceeded with extracting the height only and the width measurement provided by expert dentists. Attention U-Net achieved the highest segmentation scores compared to U-Net and UNet++, Table 1. KNN showed the best scores on all measures compared to the other models. Segmentation metrics on all models are provided in the supplementary material.

**Table 1:** Segmentation results of the lower left third molar using U-Net, attention U-Net, and UNet++ in term of Dice coefficient, Jaccard Index, Sensitivity, Specificity, Precision, and Accuracy on testing set.

Measure	U-Net			Attention U-Net			UNet++		
	Mean	Median	SD	Mean	Median	SD	Mean	Median	SD
**Dice coefficient**	0.90	0.94	0.14	**0.91**	0.94	0.14	0.83	0.88	0.17
**Jaccard Index**	0.84	0.89	0.16	**0.85**	0.89	0.15	0.74	0.79	0.17
**Sensitivity**	**0.89**	0.94	0.16	0.88	0.92	0.13	0.85	0.91	0.19
**Specificity**	0.99	0.99	0.00	**0.99**	0.99	0.00	0.99	0.99	0.00
**Precision**	0.92	0.95	0.12	**0.94**	0.97	0.12	0.84	0.86	0.14
**Accuracy**	0.99	0.99	0.00	**0.99**	0.99	0.00	0.99	0.99	0.00

**Fig 3:**
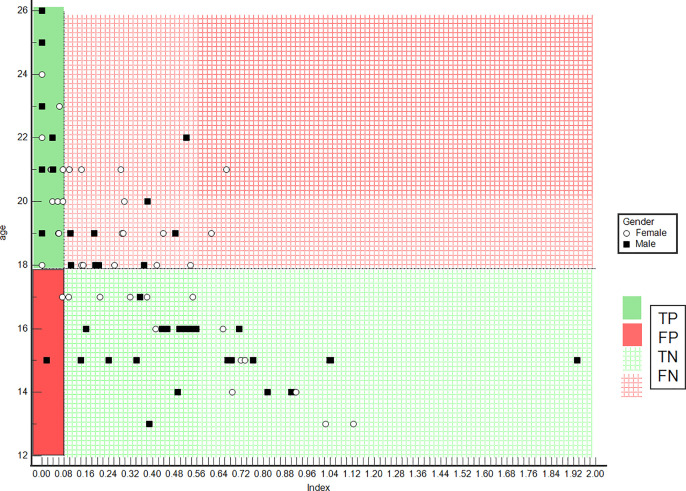
Scatter plot classification of males and females based on age and I3M. [TP: True positive, FP: false positive, TN: true negative, FN: false negative].

Comparison between the expert (dentist) and the SML (KNN) revealed that SML yielded better performance except for specificity (96% vs 100% in males and 86.7% vs 93.3% in females). The performance of both expert and SML was better for males than for females. Table 2 summarizes the performance of expert vs SML. Fig 3 shows the classification of males and females based on age and I3M. True positive represents adults classified as adults, false positive represents minors classified as adults, false negative represents adults classified as minors, and true negative represents minors classified as minors.

**Table 2:** Performance of expert vs SML in radiographic dental age estimation.

Male	Truth			Male	Truth		
**Expert**	Adult	Minor		**SML (KNN)**	Adult	Minor	
Adult	19 97.3% RT 62.1% CT 36.7% GT	0 2.7% RT 2.5% CT 1.0% GT	19 (36.5%)	Adult	21 93.7% RT 77.6% CT 45.9% GT	1 6.2% RT 7.5% CT 3.1% GT	22 (42.3%)
Minor	8 36.1% RT 37.9% CT 22.4% GT	25 63.9% RT 97.5% CT 39.8% GT	33 (63.5%)	Minor	6 26.0% RT 22.4% CT 13.3% GT	24 74.0% RT 92.5% CT 37.8% GT	30 (57.7%)
	27 (51.9%)	25 (48.1%)			27 (51.9%)	25 (48.1%)	52
Chi-squared = 27.2, DF = 1, P < 0.0001				Chi-squared = 28.4, DF = 1, P < 0.0001			
Sensitivity = 70.4% [49.8% to 86.2%]				Sensitivity = 77.77% [57.742% to 91.378%]			
Specificity = 100% [86.3% to 100%]				Specificity = 96% [79.6% to 99.9%]			
Accuracy = 84.6% [71.9% to 93.1%]				Accuracy = 86.5% [74.2% to 94.4%]			
AUC = 0.85 [0.73 to 0.93]				AUC = 0.87 [0.75 to 0.95]			
**Female**				**Female**			
**Expert**	Adult	Minor		**SML (KNN)**	Adult	Minor	
Adult	17 97.3% RT 62.1% CT 36.7% GT	1 2.7% RT 2.5% CT 1.0% GT	18 (39.1%)	Adult	24 93.7% RT 77.6% CT 45.9% GT	2 6.2% RT 7.5% CT 3.1% GT	26 (56.5%)
Minor	14 36.1% RT 37.9% CT 22.4% GT	14 63.9% RT 97.5% CT 39.8% GT	28 (60.9%)	Minor	7 26.0% RT 22.4% CT 13.3% GT	13 74.0% RT 92.5% CT 37.8% GT	20 (43.5%)
	31 (67.4%)	15 (32.6%)	46		31 (59.2%)	15 (40.8%)	46
Chi-squared = 9.6, DF = 1, P < 0.0001				Chi-squared = 16.5, DF = 1, P < 0.0001			
Sensitivity = 54.8% [36.0% to 72.7%]				Sensitivity = 77.4% [58.904% to 90.406%]			
Specificity = 93.3% [68.1% to 99.8%]				Specificity = 86.7% [59.540% to 98.342%]			
Accuracy = 67.4.53% [52.0% to 80.5%]				Accuracy = 80.4% [66.085% to 90.642%]			
AUC = 0.74 [0.59 to 0.86]				AUC = 0.82 [0.68 to 0.92]			

## Discussion

The segmentation result of Attention U-Net has shown a clear segmentation of the third molar with good prediction of tooth shape and clear edges. The U-Net showed a good prediction of the third molar location and shape, but with blurry edges. UNet++ predicted the exact location of the third molar; however, it did not show clear details like other models. The blurriness issue was handled by thresholding the result. Overall, Attention U-Net performed well in predicting the location and shape of a single object, the lower left third molar, which is surrounded by similar color intensity objects such as cancellous and cortical bones.

Several studies developed a semantic segmentation method using Attention U-net to segment retinal vessels [12–14]. They compared their method results against the U-Net with three datasets. Attention u-net achieved the highest F1, ROC, AUC, and sensitivity score. In addition, the network showed improved segmentation of boundaries and thin vessels. Attention U-Net generates smooth, better-connected streamlines, and clear extraction of waterbody features. This is due to the attention gate mechanism, which gives it the ability to capture a high level of detail that eventually results in better segmentation and fewer errors while predicting the masks.

Using multiple machine learning algorithms improves results and was best obtained from K-NN, RF, and DT [15,16]. K-NN is commonly used because of its ability to deal with a small amount of training data and has shown promising results in age estimation [17,18]. Thus, K-NN was selected as a base classifier in addition to testing multiple algorithms, including RF, DT, SVM, LDA, GB, LOGR, and MLP.

Radiographic legal age estimation using SML (using KNN) showed acceptable performance and generally scored higher compared to the expert. The SML (KNN) scored sensitivity = 77.77%, specificity = 96%, and AUC = 0.87 in males. In the female group, it scored sensitivity = 77.4%, specificity = 86.7%, and AUC = 0.82. Whereas for the expert scores were: sensitivity = 70.4%, specificity = 100%, AUC = 0.85 for male, and for female: sensitivity = 54.8%, specificity = 93.3%, AUC = 0.74. These results show that the validity of I3M for legal age in the male group is higher than in the female group. This is not in contradiction to previous reports due to wider development and hormonal variations in female maturation compared to male [2,3,19,20].

The SML (KNN) improved the sensitivity of age estimation across both genders, especially in females, where seven adults (18−21 years and I3M 0.14–0.26) were correctly classified compared to human expert indices. However, there was a slight drop in specificity performance. The three minors misclassified as adults were two females aged 17 and one male aged 16.

A meta-analysis, based on 16 studies, assessing the accuracy of the I3M to estimate the legal age of 18 years based on expert measurements, revealed an overall pooled sensitivity of 0.86 (0.84 to 0.87), a pooled specificity of 0.93 (0.92 to 0.94), and the AUC was 0.96, indicating an overall high discrimination effect. Albeit several included studies, including one on the Saudi population [2], showed suboptimal performance, as noted in the lower range in sensitivity of 0.52–1.00 and specificity range of 0.75–1.00 [21]. While it is important to correctly classify adults (sensitivity), it is the misclassification of minors as adults (specificity) that can result in their unfortunate legal treatment [2,20].

The automated methods provided improved performance, efficiency, and adaptability to new populations; however, with a wide range of accuracy ranging from 27% to 100%, sensitivity from 0.42 to 1.00, and specificity from 0.80 to 0.99 [5,22,23].

The study by Franco et al. [19] is the most similar to our study because it aimed to estimate adulthood at 18 years threshold based on third molar development. The authors combined metric and stage methods and produced a model accuracy of 0.83 (female) and 0.94 (male), specificity of 0.77 (female) and 0.93 (male), and sensitivity of 0.86 (female) and 0.90 (male). While their study produced a better classification of adults, our study was more accurate in classifying minors.

Guo et al. [24] classified the dental age (threshold 18 years) using an end-to-end (CNN) model compared with the Demirjian method. Similar to our work, the CNN outperformed the expert in sensitivity (87.7% vs 89.2–90%); however, there was a drop in specificity (95.5% vs 86.6–94.4%). Similarly, Upalananda et al. found a drop in age estimation specificity in CNN compared to experts [25]. There are no evident explanations for the underperformance in specificity. It can be related to variance in the data or the quality of radiographs. The scientific evidence is not significant regarding the use of Demirjian´s method (stage H mandibular third molar) to assess the age> or < 18 years [26]. However, when combined with other methods such as I3M, it improved the estimation performance [19].

It is important to note that by retaining human-measured apex width measurement and allowing the SML to train on height analysis, the machine was able to detect subtle cues in pixel values beyond human perception. Training the AI model on gender-balanced datasets allows automatic learning of gender-specific variation, thus improving the index validity, especially in females, and addressing the well-known challenge of how female biological and hormonal factors influence developmental variability.

A fully automated dental age estimation model with no human interference outperformed methods that manually define features, achieving higher accuracy and objectivity [18,27–29]. Recent work is producing improved CNNs for radiographic age estimation, however most work is based on Demirjian stages and not I3M, West Asia remains under-represented. Moreover, the studies rarely examine the sensitivity and specificity of classification models against experts, which is important especially in the context of binary age estimation, where misclassifying a minor can have serious implications.

## Conclusions

Based on the submitted work, Attention U-net outperformed other models in segmentation. The validity of I3M for radiographic legal age estimation using SML (KNN) was higher than manual methods by humans. However, there is a need for further improvement to avoid misclassifying minors as adults.

The shortcomings of this work include the sample size and the dependence of SML on human expert width measurements. Future work should focus on analyzing radiomic features rather than on a specific index or atlas. Such quantitative features allow the SML to mine micro-features to form accurate predictions. Multi-modal systems using several dental measures, including 3D imaging, and population or gender adaptation may bring us closer to an End-to-end system that outputs an age estimate with confidence interval.

## Supporting information

S1 File Supplementary file: Mean scores of the performance measures for DT, RF, LDA, GB, LR, SVM, KNN, and MLP classifiers. (DOCX)
