# Supplementary material for: Radiographic legal age estimation based on third molar development using Machine-learning algorithms
Source: PLoS One. 2026 Jul 30;21(7):e0354704. doi: 10.1371/journal.pone.0354704 (PMC13422845; doi:10.1371/journal.pone.0354704)
Supplement: S1 File — (DOCX) [file pone.0354704.s001.docx]

**Supplementary Table: Mean scores of the performance measures for DT, RF, LDA, GB, LR, SVM, KNN, and MLP classifiers**

| **Algorithm** | **Metrics** | **80%** | **10-Fold** |
| --- | --- | --- | --- |
|  | Accuracy | 0.82 | 0.83 |
|  | F1 | 0.80 | 0.78 |
| DT | Precision | 0.71 | 0.77 |
|  | Sensitivity | **0.93** | 0.79 |
|  | Specificity | 0.74 | 0.84 |
|  | Accuracy | 0.83 | 0.84 |
|  | F1 | 0.81 | 0.81 |
| RF | Precision | 0.73 | 0.80 |
|  | Sensitivity | **0.93** | 0.82 |
|  | Specificity | 0.76 | 0.86 |
|  | Accuracy | **0.85** | 0.84 |
|  | F1 | **0.83** | 0.78 |
| LDA | Precision | **0.77** | 0.81 |
|  | Sensitivity | 0.90 | 0.82 |
|  | Specificity | **0.81** | 0.88 |
|  | Accuracy | 0.82 | 0.86 |
|  | F1 | 0.80 | 0.82 |
| GB | Precision | 0.72 | 0.81 |
|  | Sensitivity | 0.90 | 0.82 |
|  | Specificity | 0.76 | 0.86 |
|  | Accuracy | **0.85** | 0.84 |
|  | F1 | **0.83** | 0.78 |
| LOGR | Precision | **0.77** | 0.82 |
|  | Sensitivity | 0.90 | 0.76 |
|  | Specificity | **0.81** | **0.89** |
|  | Accuracy | 0.81 | 0.84 |
|  | F1 | 0.79 | 0.79 |
| SVM | Precision | 0.70 | 0.80 |
|  | Sensitivity | 0.90 | 0.79 |
|  | Specificity | 0.74 | 0.86 |
|  | Accuracy | 0.84 | **0.87** |
|  | F1 | 0.82 | **0.83** |
| KNN | Precision | 0.74 | **0.84** |
|  | Sensitivity | **0.93** | **0.83** |
|  | Specificity | 0.78 | **0.89** |
|  | Accuracy | 0.83 | 0.85 |
|  | F1 | 0.81 | 0.81 |
| MLP | Precision | 0.73 | 0.81 |
|  | Sensitivity | **0.93** | **0.83** |
|  | Specificity | 0.76 | 0.87 |
